# A framework for testing the impact of co-infections on host gut microbiomes

**DOI:** 10.1186/s42523-022-00198-5

**Published:** 2022-08-09

**Authors:** Dominik W. Schmid, Gloria Fackelmann, Wasimuddin, Jacques Rakotondranary, Yedidya R. Ratovonamana, B. Karina Montero, Jörg U. Ganzhorn, Simone Sommer

**Affiliations:** 1 Institute of Evolutionary Ecology and Conservation Genomics, Ulm University, Ulm, Germany; 2 Faculty of Medicine, Institute for Infectious Diseases, University of Bern, Bern, Switzerland; 3 Faculté Des Sciences, Université d’Antananarivo, 566 Antananarivo, Madagascar; 4 Department of Animal Ecology and Conservation, Institute of Zoology, Universität Hamburg, Hamburg, Germany

**Keywords:** Gut microbiome, Dysbiosis, Co-infections, Parasites, Virus, Helminths, Wildlife health, Non-human primate, Disease ecology, One health

## Abstract

Parasitic infections disturb gut microbial communities beyond their natural range of variation, possibly leading to dysbiosis. Yet it remains underappreciated that most infections are accompanied by one or more co-infections and their collective impact is largely unexplored. Here we developed a framework illustrating changes to the host gut microbiome following single infections, and build on it by describing the neutral, synergistic or antagonistic impacts on microbial α- and ß-diversity expected from co-infections. We tested the framework on microbiome data from a non-human primate population co-infected with helminths and Adenovirus, and matched patterns reported in published studies to the introduced framework. In this case study, α-diversity of co-infected Malagasy mouse lemurs (*Microcebus griseorufus*) did not differ in comparison with that of singly infected or uninfected individuals, even though community composition captured with ß-diversity metrices changed significantly. Explicitly, we record stochastic changes in dispersion, a sign of dysbiosis, following the Anna-Karenina principle rather than deterministic shifts in the microbial gut community. From the literature review and our case study, neutral and synergistic impacts emerged as common outcomes from co-infections, wherein both shifts and dispersion of microbial communities following co-infections were often more severe than after a single infection alone, but microbial α-diversity was not universally altered. Important functions of the microbiome may also suffer from such heavily altered, though no less species-rich microbial community. Lastly, we pose the hypothesis that the reshuffling of host-associated microbial communities due to the impact of various, often coinciding parasitic infections may become a source of novel or zoonotic diseases.

**Supplementary Information:**

The online version contains supplementary material available at 10.1186/s42523-022-00198-5.

## Introduction

Multicellular life evolved in an environment of unicellular, microbial co-inhabitants, and is forced to interact with and, eventually, host already present microbiota [1, 2]. As a collaborative venture, hosts entrusted partial sovereignty of important functions to the microbiome, including modulation of host metabolism [3], development [4], behaviour [5] and immunity [6–8]. The gut microbiome takes up many of these tasks. A high microbial diversity and constant direct and indirect molecular crosstalk between the genomes of interacting hosts, bacteria, viruses and fungi (i.e., holobiont) maintain a stable gut microbial community, optimise microbial functions and buffer against disturbances [9, 10]. The most radical changes in the commensal microbial community are often connected to macro- and microparasitic infections (e.g., viruses [11]; bacteria [12]; helminths [13], fungi [14]).

**Fig. 1:**
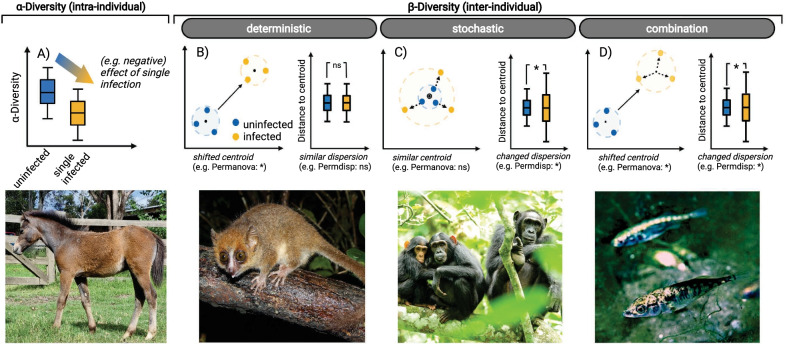
The impact of single infections on α- and ß-diversity of the host’s microbiome with examples. **A** Single infections can have a directional effect on microbial species diversity. Equine gut microbial α-diversity, for instance, decreased following helminth infection [17]. **B** Single infections may result in deterministic changes to the microbial community composition (i.e., ß-diversity), which are characterized by a shift of the centroid (= black dot; e.g., analysed by Permutational Multivariate Analysis of Variance, Permanova). In this case, the dispersion stays similar (e.g. analysed by Permutational Analysis of Multivariate Dispersions, Permdisp). For example, the gut microbial composition shifted in Adenovirus-infected mouse lemurs [11]. **C** Alternatively, single infections may lead to a changed dispersion, which can be visualized as distance to centroid (spread = black arrow). An example are chimpanzees infected by the simian immunodeficiency virus, which had a more dispersed gut microbiome [21]. **D** Single infections can also lead to both stochastic and deterministic effects. Three-spined stickleback (*Gasterosteus aculeatus*), for instance, infected with the cestode *Schistocephalus solidus* had a more dispersed and shifted gut microbial community [22]. * = significant differences (i.e., *p*-value < 0.05); ns = non-significant differences (i.e., *p*-value > 0.05)

Parasites exploit unused metabolic products, induce inflammation or compete for space and resources with commensal bacteria [15, 16]. As a consequence, parasites may transform a microbial community into a disturbed or dysbiotic microbiome (i.e., pathobiome) benchmarked by changes in intra-individual microbial diversity (i.e., α-diversity), shifts or dispersal inter-individual diversity (i.e., ß-diversity) and overdominance or disappearance of specific bacterial taxa (conceptualised in Fig. 1; [9]). Helminth-infections, for example, modulate the abundance of immune-regulatory commensals in horses (Fig. 1A; [17]), while infections with the human immunodeficiency virus (HIV) lower overall gut microbial richness and evenness [18, 19]. In wild populations of Malagasy mouse lemurs (*Microcebus griseorufus*), inter-individual similarity in microbial composition differed between uninfected and Adenovirus^+^ individuals (Fig. 1B; [11, 20]). The microbiomes of chimpanzees infected with the Simian immunodeficiency virus were more dissimilar between infected individuals compared with uninfected hosts (Fig. 1C; [21]).

In turn, an infection can undermine microbe-mediated, metabolic or immunological functions and facilitate further infections [23–26]. The protozoan *Toxoplasma gondii*, for instance, repeatedly caused declines in ASVs (amplicon sequence variants) of the family *Proteobacteria* and *Bacteroidetes* in a murine model [27]. *Bacteroidetes* are key to inducing inflammation responses and counter infections. SARS-CoV-2—the latest installment of a series of viruses with pandemic potential and zoonotic origin—was documented to shift the host gut microbiome community and allow opportunistic and pathogenic bacteria to take over [28, 29]. Importantly, however, commensals are not defenseless against parasitic invaders [10]. In the case of patients with COVID-19, four commensal *Bacteroides* species were found to downregulate the expression of the ACE2 receptor, which is used by the disease agent SARS-CoV-2 to enter host cells [28]. Disturbances of the microbial community may be symptomatic of an unhealthy host or a reaction to optimize immune-regulatory functions against an invader [9], but shifts away from homeostasis may invite secondary infections to take hold. In brief, the interaction between the host microbiome and an infection undoubtably contributes to disease severity, progression and recovery [30].

Yet our knowledge about the impact of parasitic infections on the microbiome largely stems from single infections, aiming to link cause and effect. Little attention has been paid to the impact of co-infections even though they are the norm in nature [24, 25]: for instance, 46% of all bank voles (*Myodes glarolus*) infected with the tick-borne bacterium “*Candidatus* Neoehrlichia mikurensis” were co-infected with the zoonotic Lyme disease agent *Borrelia afyelii* [31]; and 79% of field voles (*Microtus agrestis*) were co-infected with a protozoan, virus and/or bacteria [32]; 72% of helminth-infected marbled spinefoot rabbit fish *Siganus rivulatus* were also infected with at least one additional helminth species [33]; viral co-infections in virus-positive bats range from approximately 1% [34] to up to 40% [35]. In humans, co-infections are conservatively estimated at 30%, even though some estimates extend as high as 80% for some communities [36]. For example, co-infections with HIV and hepatitis B virus range between 3 and 25% [37], and even triple infections with bacteria that use shared transmission pathways, like syphilis, reach a prevalence as high as 30% in some developed nations [38]. The occurrence of co-infections is probably underestimated since targeted detection approaches based on a priori expectations likely overlook unknown or unexpected parasites [39].

Given the importance of microbiome homeostasis for host health [6–8], changes to the microbiome arising from single infections alone, the universality of co-infections in nature, and the increased infection risk of hosts in anthropogenically changed habitats [30, 40], investigating the impact of co-infections on the host microbiome is particularly timely [9, 11]. This review begins with sketching out a framework for expected impacts of co-infections on host microbial communities. We draw examples from an extensive literature search and embed the empirical findings in our framework. Moreover, we re-analysed a published data set from a wild population of Malagasy mouse lemurs (*M. griseorufus*; [11, 20]) under inclusion of co-infection information and align the findings with our framework, adding to the few published examples. Finally, we critically dissect limitations, point to unanswered questions and frame the importance of co-infection research in the context of disease ecology and One health considerations.

## Expected impacts of co-infections on microbial communities: a theoretical framework

Broadly speaking, different parasites infecting the same host can assist, counter or disregard one another in their impact: some helminths, for instance, suppress the host’s inflammatory responses, which favors the establishment and rapid growth of micro- or macroparasites [24–26]. Wild rabbits infected with the helminth *Trichostrongylus retortaeformis*, but not with the helminth *Graphidium strigosum,* experienced greater infection intensity when co-infected with the immunosuppressive myxoma virus [41]. Other parasites compete for limited host resources [24–26], resulting in negative correlations between the abundance of co-infecting parasites. Such inverse relationships were found in domestic sheep [42] and wild mice (genus: *Peromyscus*) [43] infected with the protozoan *Eimeria* and *Campylobacter* or helminths, respectively. Some co-infecting parasites even actively offset the development and manipulation of other parasites to advance their own survival and transmission [44]. In any case, parasite-specific traits likely govern these parasite-parasite interactions [24, 26]. Hence, trait-mediated effects are equally likely to determine parasite-parasite-microbiome interactions and, thus, the impact of co-infections on host microbiome stability.

This means multiple infections are predicted to alter the host’s microbiome in their own specific way. In recent years, high-throughput 16S ribosomal RNA amplicon and shotgun sequencing data pushed the study of microbial communities to a new era. The ability to look at community patterns rather than just specific taxa alone also meant that new and old analytical tools, regularly employed by community ecologists, were now available to microbiologists and that these observations and patterns are now embedded in rich ecological theory adapted for microbial communities (e.g., keystone species; α/ß-diversity; Anna-Karenina principle; reviewed in [15, 45–47]). And yet, a cohesive framework of theoretical predictions outlining how co-infections could impact gut microbial communities is currently lacking.

**Fig. 2:**
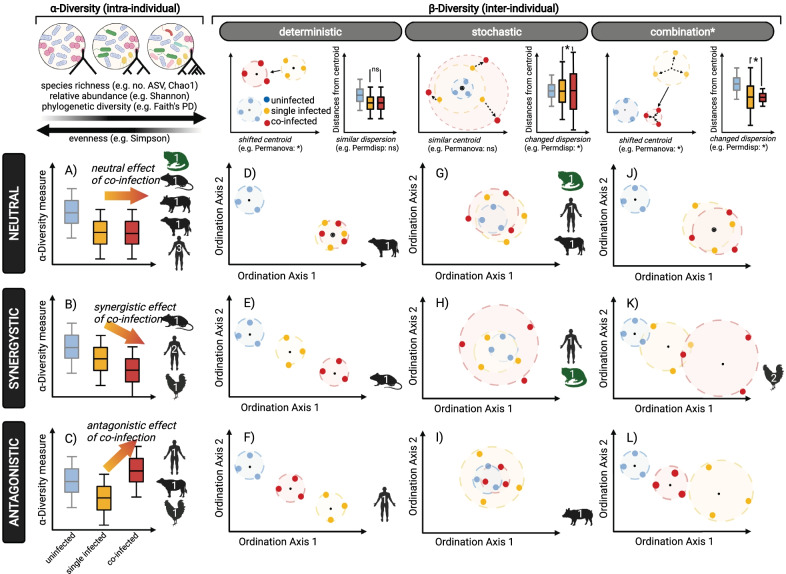
A framework to assess the impact of co-infections on α- and ß-diversity of the host’s microbiome. The top banner provides an overview of different α-diversity metrics, and the patterns created by a shift in centroid (i.e., a deterministic effect with centroid = black dot) or when plotted as distance from centroid (i.e., a stochastic effect with spread = black arrow), both describing ß-diversity. Based on the impact a single infection (yellow) has on uninfected hosts (blue), the effect of a co-infection (red) can be classified as either neutral, synergistic or antagonistic. Animal symbols are in reference to the focal organism of studies featured in Table 1 and the number represents the frequency a similar result was found. * = significant differences (i.e., *p*-value < 0.05); ns = non-significant differences (i.e., *p*-value > 0.05)

Our framework builds on these ideas (Fig. 2). Specifically, we developed testable predictions of the impact of co-infections using common community and diversity metrices. The null hypothesis to be tested follows the same principle as for single infections (Fig. 1 i.e., α-diversity of uninfected group does not change following single infections), namely that a co-infection does not alter the diversity of the gut microbial community when compared to the singly infected reference groups (Fig. 2). Importantly though, the impact of co-infections on the microbiome must primarily be levelled against that of the single infections. Hence, when compared to the effect of single infections on the host microbiome, co-infections can either be neutral, synergistic or antagonistic. However, the choice of reference markedly influenced the outcome, meaning that for observational studies individuals with single infections of either pathogen should be compared against the co-infected group (see “Case study: Neutral and synergistic effects of a co-infection on the gut microbiome of a non-human primate” section), while for experimental studies the sequence of infection determines which group represents the reference. In the following we outline our framework in more detail and showcase empirical evidence from 14 studies (Table 1; selected from 397 that fit the search criteria; see Additional file for details on the systematic literature search; Additional file 1: Fig. S1, Table S1) that report and compare the impact of single and co-infections.

**Table 1:** Summary table of research detailing the effect of co-infections on the level of dysbiosis in host guts

Host	Reference-infectant	Co-infectant	Level of dysbiosis					
			Taxa abundance	α-diversity	Impact	ß*-*diversity	Impact	References
*Veterinary research*								
Domestic pig (*Sus scrofa domesticus)*	Bacterium (*Salmonella enterica* serovar Typhimurium)	Bacterium (*Lawsonia intracellularis*)	↓ *Clostridium* ↑ *Lactobacillus*	~Evenness (Simpson), richness (Chao1, ASVs)	N, A	Homogeneous clustering	A	[48]
Domestic chicken *(Gallus gallus domesticus)*	Protozoan (*Histomonas meleagridis*)	Bacterium (Avian pathogenic *E. coli)*	↓ *Lactobacillus*, *Faecalibacterium, Ruminococcaceae*; ↑ *Escherichia*, *Bacteroides*, *Fusobacterium*, *Helicobacter*	↓ Richness (Chao1) and diversity (Shannon)	S	↑ Heterogeneous clustering	S	[49]
Domestic chicken *(G. gallus domesticus)*	Virus (Avian leukosis virus-J)	Virus (Marek’s disease virus; Avian reticuloendotheliosis virus)	↑ *Bacteroides, Enterococcus*	↑ Richness (Chao1); ~ Diversity* (Shannon)	S	↑ Heterogeneous clustering	S	[50]
*Experimental laboratory research*								
Lab mouse (*Mus musculus*—INS-GAS)	Helminth (*Heligmosomoides polygyrus*)	Bacterium *(Helicobacter pylori)*	↓ colonisation of Altered Schaedler Flora 356	*Na*	*Na*	*na*	*na*	[51]
Lab mouse (*M. musculus*—C57BL/6 WT Stat6^±^*)*	Helminth (*Trichinella spiralis*)	Virus (murine norovirus MNV CW3)	↓ *Turicibacteraceae*; ↑ *Lactobacillacaeae, Clostridiales*	*Na*	*Na*	*na*	*na*	[52]
Lab mouse (*M. musculus*—C57B1/6)	Protozoan (*Giardia lamblia*)	Bacterium (*E. coli*)	↑ *Enterobacteriaceae*	*Na*	*Na*	*na*	*na*	[53]
Lab mouse (*M. musculus*—C57BL/6)	Bacterium (*Campylobacter jejuni*)	Protozoan (*Toxoplasma gondii*), Bacteria (*S. typhimurium*; entero-invasive *E. coli*; *Listeria monocytogenes*)	↑ *Enterobacteriaceae* in *C. jejuni*/*T. gondii* and *C. jejuni*/*S. typhimurium* coinfected mice; ↓ *Clostridiales* and *Lachnospiraceae* in all co-infected groups; ↑ *Dorea* and unclassified S24-7 in *C. jejuni*/*E. coli*	~ /↓ Richness (Chao1) and diversity (Shannon)	N, A	*na*	*na*	[54]
Lab mouse (*M. musculus*—SPF ICR)	Protozoan (*T. gondii*)	Helminth (*Schistosoma japonicum*)	↑ *Clostridiaceae*	↓ Richness (Chao1); ~ diversity (Shannon);	S	↑ Heterogeneous clustering	S	[55]
*Medical research*								
Human (*Homo sapiens*)	Norovirus (NV)	Bacteria (Enterotoxigenic *E. coli*)	↑ *Bacteroides*	~ Richness (Chao1), evenness (Simpson)	N	↑ Heterogeneous clustering than single infections	N	[56]
Human (*Homo sapiens*)	Norovirus (NV) or Rotavirus (RV)	Bacteria (Enteroaggregative or enteropathogenic *E. coli*)	↓ *Enterococcus* and *Veillonellaceae* in NV-*E. coli* co-infected; In RV infected, *Lachnospiraceae* and *Collinsella* were differentially abundant dep. on *E. coli* co-infectant	~ Diversity (Shannon) for NV-co-infected; ↓ diversity only for RV + EAEC	N, S	*na*	*na*	[57]
Human (*H. sapiens*)	Protozoan (*Plasmodium vivax*)	Helminths (e.g., *Trichuris trichiura*)	↓ *Bacteroides;* ↑ *Prevotella copri, Clostridiaceae*	~ Diversity (Shannon)	N	Homogeneous clustering	N, A	[58]
Human (*H. sapiens*)	Eukaryote (*Giardia duodenalis*)	Helminths (e.g., *Ascaris lumbricoides*)	↑ *Prevotella* (e.g., *Prevotella copri*) compared with helminth single infected group	~ Diversity (Shannon) to uninfected, but ↑ than *G. duodenalis* and ↓ than helminth singly-infected	A	*na*	*na*	[59]
Human (*H. sapiens*)	Virus (Human immunodeficiency virus)	Virus (Hepatitis C virus)	-	↓ Diversity (Shannon)	S	↑ ß*-*diversity distances	S	[60]
*Wildlife research*								
African buffalo *(Syncerus caffer)*	Bacterium *(Mycobacterium bovis*)	Nematodes (e.g., *Cooperia fuelleborni*)	↓ SHD-231	~ Diversity (Shannon), evenness (Pielou), OTU richness	N	~ ß*-*diversity distances	N	[61]

The identity of the host and co-infecting parasite species is shown in relation to the impacts reported, starting with changes in taxa abundance, α-diversity and ß-diversity. Diversity metrices were assessed based on the nature of change seen following co-infection (i.e., neutral, synergistic, antagonistic) in relation to the effect observed after a single infection (with the reference infectant). Details on the literature survey and references are provided in the Additional file, Additional file 1: Fig. S1, Table S1)

*na* signifies a lack of information*;* impacts: N = neutral, S = synergistic, A = antagonistic; ↑ = increase; ↓ = decrease; ~  = comparable with single infection unless otherwise specified

### Neutral effects

If a single infection does not result in observable changes to the gut microbiome compared to the uninfected group, then the effect is negligible or neutral. For instance, the microbial α-diversity—a measure of intra-individual species richness, evenness and phylogenetic diversity—of mouse lemurs infected with an Adenovirus (AdV) was indistinguishable from uninfected individuals [11]. Similarly, and by extension, if a co-infection does not alter the gut microbiome beyond the change that a single infection caused i.e., when both the single and co-infection cause the same observable changes to the gut microbiome, then the effect of the co-infection is said to be neutral (Fig. 2A, D, G, J). In other words, neutral impacts emerge when one co-infecting parasite does not interfere with the manipulation of the other. Neutral effects can be observed when considering both α- and ß*-*diversity. Whilst α-diversity reflects intra-individual diversity, ß*-*diversity is a measure of inter-individual diversity in microbial communities. Impacts on the latter can be investigated in two different ways: by testing for deterministic effects, which move the microbiome communities towards a different but consistent configuration (i.e., shifted centroid location in ordination space), or stochastic effects (i.e., Anna-Karenina principle [AKP]), which translate into unique configurations of each individual microbial community (i.e., changed dispersion from a common group centroid; Figs. 1 and 2 top banner).

Neutral effects of additional infections on gut microbial α-diversity were demonstrated in at least one experimental murine study [54], a study on domestic pigs [48] and in several medical studies on humans ([56–58], Table 1, Fig. 2A). Neutral effects are also present among wild animals. In a free-ranging African buffalo (*Syncerus caffer*) population infected with both bovine tuberculosis (*Mycobacterium bovis,* TB) and gastrointestinal helminths, gut microbial α-diversity differed only between uninfected and helminth-infected, but TB-negative buffalos [61]. Microbial richness of co-infected buffalos was indistinguishable from buffalos either only TB-positive or only helminth infected (Fig. 2A). Thus, the effect of TB infection on α-diversity was neutral with respect to the change seen in buffalos solely infected with helminths. Additionally, the study finds the gut microbial community composition to remain unaffected by co-infections compared to the single infections status (Fig. 2D, G; [61]). Lastly, this work highlights some of the potential from wildlife research in the context of co-infections (Box 1). Taken together, neutral impacts might be a common outcome of co-infections (Table 1).

BOX 1: Tracking changes in microbiomes from wild animals is difficult but feasible Information about the impact of co-infections on the microbiome of wild animals, in particular, are scarce, not least because a diverse set of skills is needed to capture and analyse a variety of data (e.g., intensive capture-recapture efforts in the field; molecular screening for parasites and microbes in the laboratory; -omics pipelines for data processing and analysis). A possible source of more information are studies primarily dedicated to screening vectors for diseases with zoonotic potential. Since species richness predicts parasite diversity, highly diverse vector groups are targeted by a higher number of parasites than less diverse taxa [62]. Rodents, bats, primates and birds spearhead the list of diverse zoonotic vectors and reservoirs with regular interactions with humans as we further encroach on their natural habitats [63, 64]. Moreover, these taxa are often highly co-infected [31, 35, 65–67]. Infections in wildlife are likely so common that an uninfected state may prove itself to be a rare circumstance [65]. These vectors and reservoirs likely transmit a plethora of parasites to new hosts (following the 80:20 rule of disease transmission [68]). Gregarious bats, for instance, often carry a variety of viral strains (e.g. [34, 35].) and have been implicated as reservoirs of SARS-like CoVs [69] and sources of SARS-CoV-2 [70]. Yet, fecal samples are easy to collect from trapped rodents and netted bats and birds (e.g., [71, 72]), and tracking of vector populations on a regular basis would allow gathering of important temporal data on infection status and microbiome diversity [15, 73]. Even perturbation studies aiming to vaccinate or treat against certain parasites can be a powerful, even if laborious endeavor and bring a more experimental approach to wildlife research [61]. Importantly, more detailed infection information will likely require more nuanced analytical approaches to deal with parasite-specific and infection load related impacts, possibly also not easily captured by diversity metrices. Vectors and reservoirs are ideal natural model systems to study such dense infection information and the subsequent impact of co-infections on host microbiomes.

### Synergistic effects

As an alternative to neutral effects, single infections can increase or decrease microbial α-diversity and change centroid position and/or dispersion patterns of ß*-*diversity (Fig. 1). A single infections with HIV, for instance, can lower gut microbial richness and evenness [18, 19]. However, if a co-infection compounds the changes caused by a single infection even further, then the effect can be either synergistic or antagonistic, depending on the direction of these changes. In the case of synergistic effects, the impact of a co-infection is greater than the measured effect of a single infection alone. For example, if a single infection reduces (or increases) α-diversity, then a co-infection would further reduce (or further increase) gut microbial α-diversity (Fig. 2B). Such synergistic effect was showcased in a study on mice infected with the protozoan *Guardia lamblia* and an enteroaggregative *E. coli* (Table 1; [53]). The abundance of *Enterobacteriaceae* increased following co-infection. As a consequence, co-infection altered microbiome functionality (e.g., muscle metabolism and energy expenditure regulation governed by the creatine:creatinine ratio and nicotinamide pathways, respectively) [53].

The same prediction can also be formulated for ß-diversity. When looking at deterministic effects, shifts in a gut microbial community structure following a single infection could be accentuated during co-infection (Fig. 2E). Besides further reductions in microbial α-diversity, mice experimentally co-infected with *Schistosoma japonicum* and *T. gondii* displayed such a shift in microbiome composition further along ordination axis 1 than found in any of the singly infected groups (Table 1; [55]). Both parasites are known to change the host microbiome independently from one another [27, 55, 74, 75]. The helminth *S. japonicum* causes schistosomiasis leading eventually to periportal and liver cirrhosis. Schistosomiasis-induced alteration of the microbiome composition in murine [74] and human hosts [75] is thought to underlie changes in Th1/Th2 responses linked to the development of hepatic fibrosis [55]. Yet, rather than worsening host health, the shift in diversity and composition in co-infected mice likely ameliorated *S. japonicum*-induced liver fibrosis, presumably due to the promotion of Th1 immune responses by *T. gondii* [55, 76].

When looking at stochastic effects, an increased (or decreased) community dispersion following a single infection could become more pronounced during co-infection (Fig. 2H). On their own, infections with HIV [18] and the hepatitis C virus (HCV) [77] result in more dysbiotic guts. HIV-HCV co-infected individuals experience significantly lower α-diversity (Fig. 2B) and a more pronounced ß*-*diversity dispersion (Fig. 2H), indicating fewer similarities between individual microbial communities, compared with HIV singly infected patients [60]. Moreover, the observational study found all groups to differ in their metabolome with the co-infected group being most dissimilar [60]. Collectively, these findings suggest synergism driving the dispersion in co-infected patients.

Lastly, since each parasite may induce either deterministic or stochastic changes in the microbial ß-diversity community, co-infections might yield mixed results (i.e., a shifted and more/less dispersed community; Fig. 2K). The only evidence for this comes from experimental studies on poultry (Table 1). For instance, *Histomonas meleagridis* causes histomonosis in poultry [78]. The protozoan compromises the intestinal mucosal barrier of its host, disrupting nutrient uptake and enabling the establishment of other pathogens, such as the avian pathogenic *E. coli*. In domestic chickens (*Gallus gallus domesticus*) co-infected with *H. meleagridis* and an avian pathogenic *E. coli* strain, ß-diversity was both more dispersed and shifted in co-infected pullets, while *E. coli* singly infected hosts clustered tightly together [49]. The co-infection with *H. meleagridis* also reduced the abundance of commensal bacteria, such as the *Ruminococcaceae*, which are involved in the breakdown and conversion of feed to body weight, hence co-infected chicken lost significantly more weight over the course of the experiment [49]. In both poultry studies, co-infections also favored the establishment of unique compositions of competitive, pathogenic bacteria, more distinct than those found in singly infected individuals [49, 50]. Such compositional reshuffling following frequent infections and aggressive anti-microbial treatments on animal farms has led, and is likely to lead, to the emergence of new potentially pathogenic bacteria [63, 79].

### Antagonistic effects

In contrast to synergistic effects, co-infections can counteract the effects of a single infection seemingly to return the gut microbial α- or ß-diversity (closer) toward its uninfected state (Fig. 2C, F, I, L). Such an effect can be described as antagonistic and likely evolved in some parasites as a mechanism to modulate host- and microbiome-mediated immunity in order to protect itself (and by extension its host) from a co-colonizing competitor [26]. It is important to note here that antagonistic effects are marked by a change in direction rather than entirely nullifying the effect of a single infection. Co-infections with helminths are likely prime candidates to observe antagonistic effects owing to their ability to modulate inflammatory responses and maintain gut homeostasis for their own benefit [80]. A cross-sectional study on 37 children from rural parts of Argentina co-infected with the eukaryotic protozoan *Giardia duodenalis* and helminths found antagonistic effects of the co-infection compared to single infections with either parasite: co-infected children showed higher microbiome α-diversity than *G. duodenalis* singly infected children, but lower diversity than those children only infected with helminths [59]. Yet, α-diversity of co-infected children was actually comparable to uninfected infants [59]. This indicates counteractive microbiome modification by either parasite (Fig. 2C). Critically though, the microbiomes of co-infected children lost the ability to biosynthesize Vitamin B_12_ in sufficient quantities, possibly as a result of a shifting microbiome composition with anaerobic *Prevotella* becoming the leading taxa in the *G. duadenalis* singly and co-infected group [59]. The loss of an obligatory bacteria-derived micronutrient may explain some of the pathologies (e.g. malabsorption, diarrhea) observed with *G. duodenalis* infections [81], but also showcases that, even though co-infections resulted in microbial α-diversity akin to that of uninfected children, changes in microbial composition associated with a single disease agent can transform microbiome functions with debilitating consequences for the host.

Antagonistic impacts of co-infection on the microbiome were also investigated in an observational study on 130 Columbian children infected with the malaria parasite *Plasmodium vivax* and either of two common helminths (*Trichuris trichiura*, *Ascaris lumbricoides*; [58]). While microbiome α-diversity was similar between un-, singly and co-infected children, the microbiome composition differed deterministically (Fig. 2F): *Prevotella copri* and *Clostridiaceae* were less abundant, whereas *Bacteroides* were more common in individuals only infected by *P. vivax* than in uninfected, helminth-only or co-infected children [58]. The findings indicate modulation by the helminth to maintain microbiome homeostasis, but equally suggests strong impacts of single infections with *P. vivax*. Interestingly, singly and co-infected individuals with *P. vivax* still had altered immunological parameters such as interleukins and hematocrit, indicating that *P. vivax* likely modulates immunity directly rather than via the gut microbiome.

## Case study: neutral and synergistic effects of a co-infection on the gut microbiome of a non-human primate

Non-human primates pose a significant zoonotic risk to humans owing to a more recent, shared evolutionary past [82] and an overlap in habitats following increased human encroachment [83–85]. Zoonotic Adenoviruses (AdVs), for instance, which can cause diarrhoea and mild to severe diseases in humans and other primates [86], originate more often than expected from primates [62]. Similarly, 20% of primate-borne helminths, although much more host specific, are estimated to also infect humans [87]. Monitoring programs make wild non-human primate populations a natural model system to explore the impact of infections, including co-infections, on the host’s microbiome [88]. In order to emphasise this point, we re-analysed microbiome data from helminth and AdV-infected Malagasy mouse lemurs [11, 20].

**Fig. 3:**
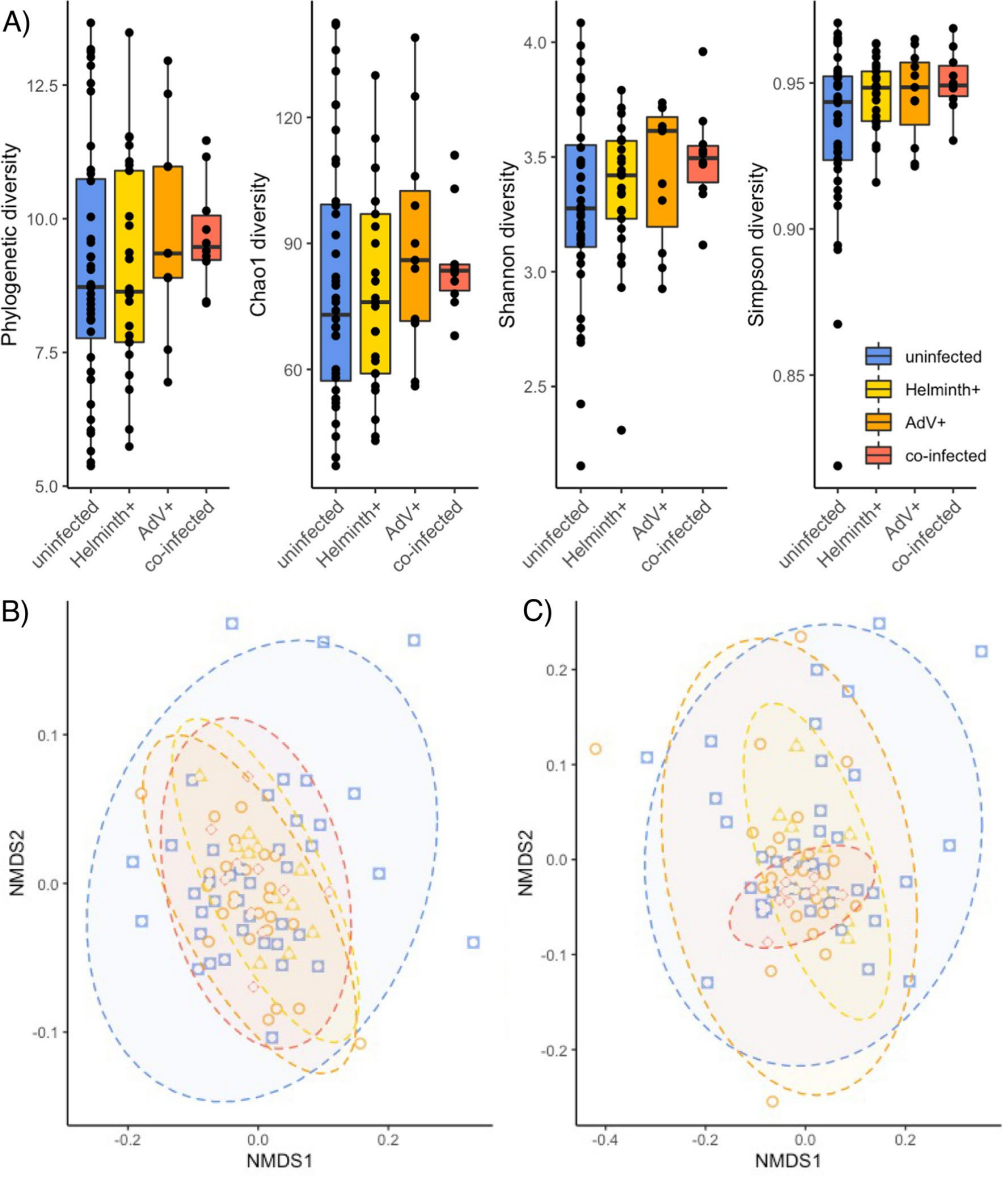
Differences in gut microbial α- and ß*-*diversity in uninfected, single-infected and co-infected mouse lemurs (*M. griseorufus*). **A** α-diversity measured by Faith’s phylogenetic diversity, Chao1, Shannon diversity, Simpson (left to right) and **B** ß*-*diversity measured by weighted and **C** unweighted UniFrac distances and illustrated by non-metric multi-dimensional (NMDS) ordination plots. Displayed are uninfected (blue squares), single-infected (helminth^+^: yellow triangle, AdV^+^: orange circles) and co-infected (red diamonds) mouse lemurs

The raw microbiome sequence data was accessed from the NCBI database (BioProject: PRJNA715730) for 86 samples with known infection status and processed using the same bioinformatics pipeline as described in [20]. The metadata revealed that a total of 40 samples were uninfected hosts, while 11 were AdV^+^, 25 helminth^+^ and 10 infected with both parasites. Like AdV infection, helminth infection represents a binomial variable without information on helminth species or infection load. Quality analysis following the Qiime 2 (version 2020.8) pipeline recovered an average of 43,070 (range 22,150–102,421) reads per sample after taxonomic assignments using the Silva classifier (v138; [89]). After applying a 0.1% prevalence threshold [45], the gut microbial community of the mouse lemurs was dominated by 34.5% Bacteroidota, 28.9% Actinobacteriota, 25.7% Firmicutes, followed by 4.6% Campilobacterota and Proteobacteria (< 2% Patescibacteria, Fusobacteriota, Cyanobacteria and Spirochaetota), which was coherent with previous findings [11]. Investigating the effect of infection status (i.e., uninfected, helminth^+^, AdV^+^ or co-infected) on four common α-diversity metrices (i.e., Faith’s phylogenetic diversity, Chao1, Shannon diversity, Simpson) consistenly yielded no differences in α-diversity across infection groups (Analysis of Variance: F_3,82_ = 0.616–1.585; *p* > 0.05, Fig. 3A). This is in line with one study showing no difference in α-diversity between AdV^+^ and AdV^−^ mouse lemurs [11], but in contrast with another study using 143 AdV-tested lemurs [20], which reported an increase in Faith’s phylogenetic diversity in AdV^+^ individuals. Yet collectively these results suggests a neutral impact of co-infection (Fig. 2A). Interestingly though, the variance between the groups differed for Faith’s phylogenetic diversity (Bartlett-test: *p* = 0.029), Chao1 (Bartlett-test: *p* = 0.022) and Simpson (Flinger-Killeen-test: *p* = 0.049), suggesting less variation in the singly and co-infected groups, despite their lower sample size.

To differentiate deterministic from stochastic effects, we compared the effect of infection status using PERMANOVAs (i.e., shifted centroid position) and PERMDISPs (i.e., altered dispersion) based on two ß*-*diversity metrices (unweighted and weighted UniFrac, [90]). Whereas no difference in centroid position was apparent among infected groups (PERMANOVAs—weighted UniFrac: F_3,82_ = 0.855, R^2^ = 0.03, *p* = 0.606; unweighted UniFrac: F_3,82_ = 1.274, R^2^ = 0.04, *p* = 0.122), gut microbial ß*-*diversity was differently dispersed (PERMDISPs—weighted UniFrac: F_3,82_ = 4.527, *p* = 0.008; unweighted UniFrac: F_3,82_ = 6.189, *p* = 0.002). Pair-wise comparisons revealed that, based on weighted UniFrac distances, all infected groups were significantly less dispersed than the uninfected group (Helminth^+^: *p* = 0.014; AdV^+^: *p* = 0.030; co-infected: *p* = 0.027), but did not differ significantly from one another in terms of their structural composition of ASVs (Fig. 3B; Additional file 1: Table S2). Unweighted UniFrac distances drew a different picture (Fig. 3C): here the gut microbiome community composition of AdV^+^ and co-infected individuals differed from uninfected ones (AdV^+^: *p* = 0.012; co-infected: *p* = 0.002), while Helminth^+^ were not dissimilar to the microbiomes from uninfected individuals (Helminth^+^: *p* = 0.144). Crucially though, singly infected groups differed compared to the co-infected group (Helminth^+^ vs. co-infected: *p* = 0.014; AdV^+^ vs. co-infected: *p* = 0.069). Collectively, these results rule out a deterministic shift of the mouse lemur gut microbiome, but suggest a rather stochastic contraction following anti-AKP expectations [15]. In short, gut microbial communities became less dispersed and, thus, more similar following a co-infection. Anti-AKP dynamics, in this context, describe precisely the opposite pattern to a frequent observation among microbiologists: ‘all healthy microbiomes are similar; each dysbiotic microbiome is dysbiotic in its own way’ (in reference to the opening line of Tolstoy’s *Anna Karenina*: ‘all happy families are alike; each unhappy family is unhappy in its own way’ [15]). Yet both are feasible outcomes: AKP, anti-AKP and non-AKP (possibly deterministic) effects were found to be as common as 50%, 25% and 25% in humans suffering from microbiome-associated diseases [91].

To sum up, both neutral (Fig. 2G) and synergistic (Fig. 2H) effects of a co-infection were observed: the AdV + infections, for instance, seem to shrink the microbiome irrespective of a co-infecting helminth, whereas a single infection with a helminth somewhat maintains similarities with a healthy gut microbial composition and only contracts to become more homogenous with the addition of the co-infecting virus. In this wild population, co-infecting parasites, therefore, shape the host’s microbiome in a way that no longer resembles its uninfected configuration.

## Discussion

Parasitic infections are a constant in nature. But rather than linking single parasites with a single disease or pathology as proposed by early pioneers of microbiology, modern disease ecology understands that the outcome of disease is largely determined by four interacting factors: the host, the parasite, the environment and the microbiome - the most recent addition [30]. As extension to the former disease triangle [92], the concept of the disease pyramid captures the direct and indirect four-way interactions between habitat disturbance, host susceptibility, pathogenicity and microbiome stability [30] and helps explain a rise in emerging or re-emerging infectious diseases in wildlife [93, 94], which in part cross trans-species boundaries to become public health problems [63, 64]. Three key realizations make the microbiome an essential addition in the light of the One health framework: the host microbiome is readily and directly impacted by habitat disturbance [40], host health [95] and parasitic infections (e.g., [11, 96]), the microbiome itself shapes host health and disease progression [9, 10], and a dysbiotic microbiome is a breeding ground for potentially harmful bacteria [97, 98]. Particularly, the acquisition of pathogenic or antibiotic properties via horizontal gene transfer or de novo mutations in the pressure cooker that is a highly destabilized microbiome, is daunting for human and animal health alike.

**Fig. 4:**
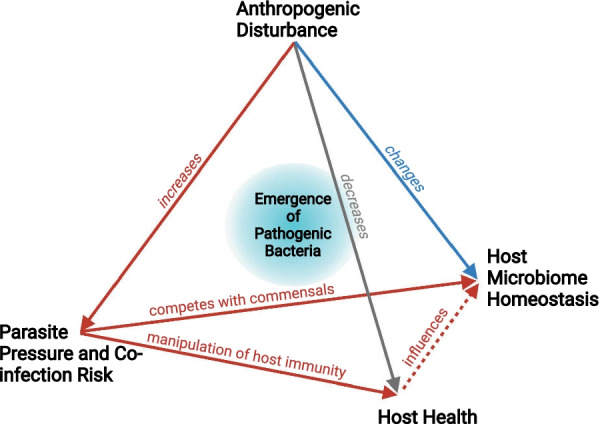
Pathways to novel, potentially pathogenic bacterial disease agents. Aside from direct changes to the host microbiome caused by habitat disturbances (blue arrows), parasites directly impact host microbiome (red solid arrow) and indirectly via manipulation of host health (red dashed arrow). Since anthropogenically disturbed habitats facilitate the transmission and persistence of parasites, direct and indirect parasite-mediated changes to a host’s microbiome may become more frequent, and a dysbiotic gut may become a source of harmful bacteria. Adapted from [30]

Parasitic infections are particularly conducive to causing microbial dysbiosis (Fig. 4). Parasites evolved to outmaneuver host-associated commensals and host defenses. As such, parasitic infections either impact host-associated microbial communities directly or indirectly via host health or the manipulation of host immunity. Distinguishing between the direct competition with host microbial communities and microbiome- or host-mediated pathways is a major challenge for future research in this field (e.g., [58, 96]). An attempt was made with an elaborate experiment on mice co-infected with the helminth *Trichinella spiralis* and a murine Norovirus [52]. Even though *T. spiralis* changed the abundance of several bacterial families independently of the co-infecting virus, the study showed empirically that compositional changes likely stem from altered interactions between the microbiome and host immunity even though only the latter was manipulated directly by the parasite [52]. Such complexity is laborious to unravel. It requires a push for novel study designs and analytical workflows to make sense of information-dense host and microbe data, which must include infection status.

Additionally, anthropogenically disturbed and biologically depauperated habitats tend to experience increased parasite diversity and prevalence (Fig. 4; e.g., [71, 99, 100]). This emphasizes the importance of understanding host-microbiome-parasite interactions. However, even information on the impact of single infections is scarce, notwithstanding findings from model organisms or pathogens with current relevance to human disease management. But single infections are not a realistic scenario, neither for wildlife (e.g., [24, 25]) nor for humans (e.g., [37, 38]) and the co-infection risk is likely to increase following further human encroachment into nature. Therefore, we hypothesize that, matching the disease pyramid [30], more pronounced anthropogenic disturbances (e.g., via habitat fragmentation, agricultural intensification, environmental pollution, climate change) coupled with increased parasite pressure likely lead to more dysbiotic host-associated microbial communities, which, in turn, may facilitate the emergence and transmission of novel and potentially pathogenic bacteria (Fig. 4).

The impact of co-infections is likely fundamental to understand these dynamics. Even from the few studies published to date, it can be said that co-infections mold how the four-way interactions pan out and host health and microbiome stability are affected. Since effects on the gut microbiome (and other microbiome communities, such as of the skin; [101]) are likely parasite-specific, it is useful to outline a predictive framework to conceptualize coupled effects (Fig. 2). In doing so, we found some instances in which co-infections altered host microbial α-diversity, but others where α-diversity seemed statistically indistinguishable from uninfected or singly-infected individuals. By contrast, ß-diversity was frequently shifted farther or dispersed more widely than after a single infection alone. Shifts in microbial communities could indicate either a cohesive (host- or microbiome-directed) response to mitigate an infection (e.g., [28]) or modulation by the parasite to enable its own establishment and persistence (e.g., [41]). Such dysbiosis may favor the persistence of harmful bacteria usually competitively excluded or down-regulated [97]. Co-infections did, for instance, often change the abundance of *Clostridiaceae* and *Bacteroides* (Table 1), both competitive bacterial families with opportunistically pathogenic members.

In contrast to deterministic shifts, dispersion following the Anna-Karenina principle may seem as if the host and/or microbiome were unable to form a coherent response to the infection stress [15]—possibly expected when encountering a novel pathogen. Alternatively, dispersion may be seen as an evolutionary strategy to uncover an effective response from more divergent microbial communities (following similar principles behind host genetic diversity in relation to selection). The results of at least one study are suggestive: the skin microbiome of the recently re-discovered neotropical Green-eyed frog (*Lithobates vibicarius*) was more divergent among those individuals encountering frequent human disturbance [102]. The disturbed skin microbiome, however, was rich in bacteria with putative inhibitory function against the chytrid fungus *Batrachochytrium dendrobatis* (*Bd*), which drove the original decline of the species. These findings offer the possibility of microbiome-mediated assistance to combat infections via a changed microbial configuration and hence function. By contrast, anti-AKP dynamics, as observed in single and even more pronounced in co-infected mouse lemurs (Fig. 3; [11, 20]), reduce variation in the microbial community to a core when the (co-)parasitic challenge is too severe. Unlike AKP, anti-AKP could thus lower the microbiome’s ability to aid host recovery. Irrespective of whether through a shift or dispersion in the microbial community, host health is expected to decline because essential microbiome-mediated functions are abandoned (e.g., [49]). As host health further deteriorates, genetic control of its microbiome is likely to suffer, accelerating “pathobiome-genesis”.

A picture emerges that co-infections may contribute to the severity of disease [30] and, as we propose here, the emergence of novel, potentially harmful bacteria concocted in a dysbiotic gut (Fig. 4). Habitats at the intercept between human- and wildlife-dominated environments and crowded with parasites materialize as breeding grounds for novel bacterial strains and, simultaneously, as research hotspots (Box 1). Recent zoonotic outbreaks and the SARS-CoV-2 global pandemic stress the interconnected nature of wildlife and human health. In fact, a global One health perspective has never been more apt. Since microbial communities are an intricate component of every ecosystem [30], their inclusion in planetary One health considerations is overdue [103]. Thus, understanding and predicting their response to challenges requires the verbalisation of a priori expectations, which we have formulated here with respect to host infection status (Fig. 2). Demystifying the black box that is the four-way interaction between environment, hosts, parasites and the microbiome will necessitate a multi-disciplinary approach from environmental, evolutionary, medical and computational scientists.

## Conclusion

Microbial ecology lacked a predictive framework outlining the possible impacts of co-infections on host microbiomes. For this reason, we introduced a cohesive framework that can be employed as a tool to test a priori expectations. Recognizing how parasites interact to shape the host microbiome may facilitate identifying patterns of (gut) microbial dysbiosis. Nevertheless, while this review is expansive, many questions remain unresolved. Most urgently, how consistent is the degree and direction of parasite-induced change to the microbiome, how do multiple infections shape commensal gut microbial communities and advance dysbiosis, and how far do feedback loops spiral and possibly threaten host health, co-inhabiting animal hosts and the encroaching human society? Understanding microbial diversity and its resilience will be of central importance for the future of the One health approach.

## Supplementary Information

**Additional file 1.** **Figure S1.** Literature search results for Set #1 (yellow), Set #2 (red), Set #3 (black dashed) and Set #4 (blue). **Table S1.** Reference list of the 14 studies included in the systematic review. **Table S2.** Pair-wise comparisons of PERMDISP results comparing the gut microbial composition of uninfected, singly infected and co-infected individuals using A) weighted UniFrac and B) unweighted UniFrac distances. Only p-values are shown.

## Data Availability

Data analysed for the case study can be found in [11, 20].

## Abbreviations

AdV: Adenovirus
AKP: Anna-Karenina principle
ASV: Amplicon sequence variant
HCV: Hepatitis C virus
HIV: Human immunodeficiency virus
HLA: Human leukocyte antigen
TB: Bovine tuberculosis
