# Supplementary material for: A framework for testing the impact of co-infections on host gut microbiomes
Source: Anim Microbiome. 2022 Aug 9;4:48. doi: 10.1186/s42523-022-00198-5 (PMC9361228; doi:10.1186/s42523-022-00198-5)
Supplement: Supplementary file 1 — Additional file 1. Figure S1. Literature search results for Set #1 (yellow), Set #2 (red), Set #3 (black dashed) and Set #4 (blue). Table S1. Reference list of the 14 studies included in the systematic review. Table S2. Pair-wise comparisons of PERMDISP results comparing the gut microbial composition of uninfected, singly infected and co-infected individuals using A) weighted UniFrac and B) unweighted UniFrac distances. Only p-values are shown. [file 42523_2022_198_MOESM1_ESM.docx]

**A framework for testing the impacts of co-infections on host gut microbiomes**

Dominik W. Schmid^1,^*, Gloria Fackelmann^1^, Wasimuddin^1,2^, Jacques Rakotondranary^3^, Yedidya R. Ratovonamana^3^, Karina Montero^4^, Jörg U. Ganzhorn^4^, Simone Sommer^1^

**Additional File 1**

**Literature assessment**

In order to corroborate our findings and assess the frequency of neutral, synergistic and antagonistic effects of co-infections on host microbiomes, we performed a systematic literature review on the Web of Science platform, on 9th August 2021. Several consecutive topic searches were used:

*Set #1: TS = (parasit* OR pathogen* OR disease OR infect* OR virus OR bacteria OR helminth OR nematode OR cestode)*

*Set #2: TS = "concomitant infections" OR "concurrent infections" OR "mixed infections" OR co-infections OR "multiple infections" OR polyparasitism OR multiparasitism) AND #1*

*Set #3: TS = (gut OR intestin* AND microbiome OR microbiota OR microbes) AND #1 AND #2*

*Set #4: TS = (gut OR intestin* AND microbiome OR microbiota OR microbes)*

Filtering for document type (article), we identified 397 primary research articles captured by the search terms (Figure S1). These articles were screened for relevance. Important criteria for inclusion and detailed evaluation were a) quantitative or qualitative analysis of microbiome data on uninfected, singly infected and co-infected individuals; b) information on at least one of the levels of microbiome dysbiosis (e.g., changed microbial abundance; altered α/ß- diversity) following co-infections; c) sufficient clarity and detail of results. From the 397 articles identified, only 14 met these criteria (Supplementary Table 1) and details on the effect of co-infections on the level of dysbiosis in host guts are listed in Table 1. Vastly different study designs and inconsistent reporting (e.g., Chao1 species richness index was only reported in 6 and Shannon diversity index only in 8 out of 14 studies) excluded the possibility of a meaningful meta-analytical approach to synthesise findings. The 14 articles grouped naturally into four categories with distinct foci, but commonalities in design and method: Veterinary research (3), experimental laboratory research (5), medical research (5) and wildlife research (1) (Table 1).

**Additional Figure**

**Fig. S1**

**
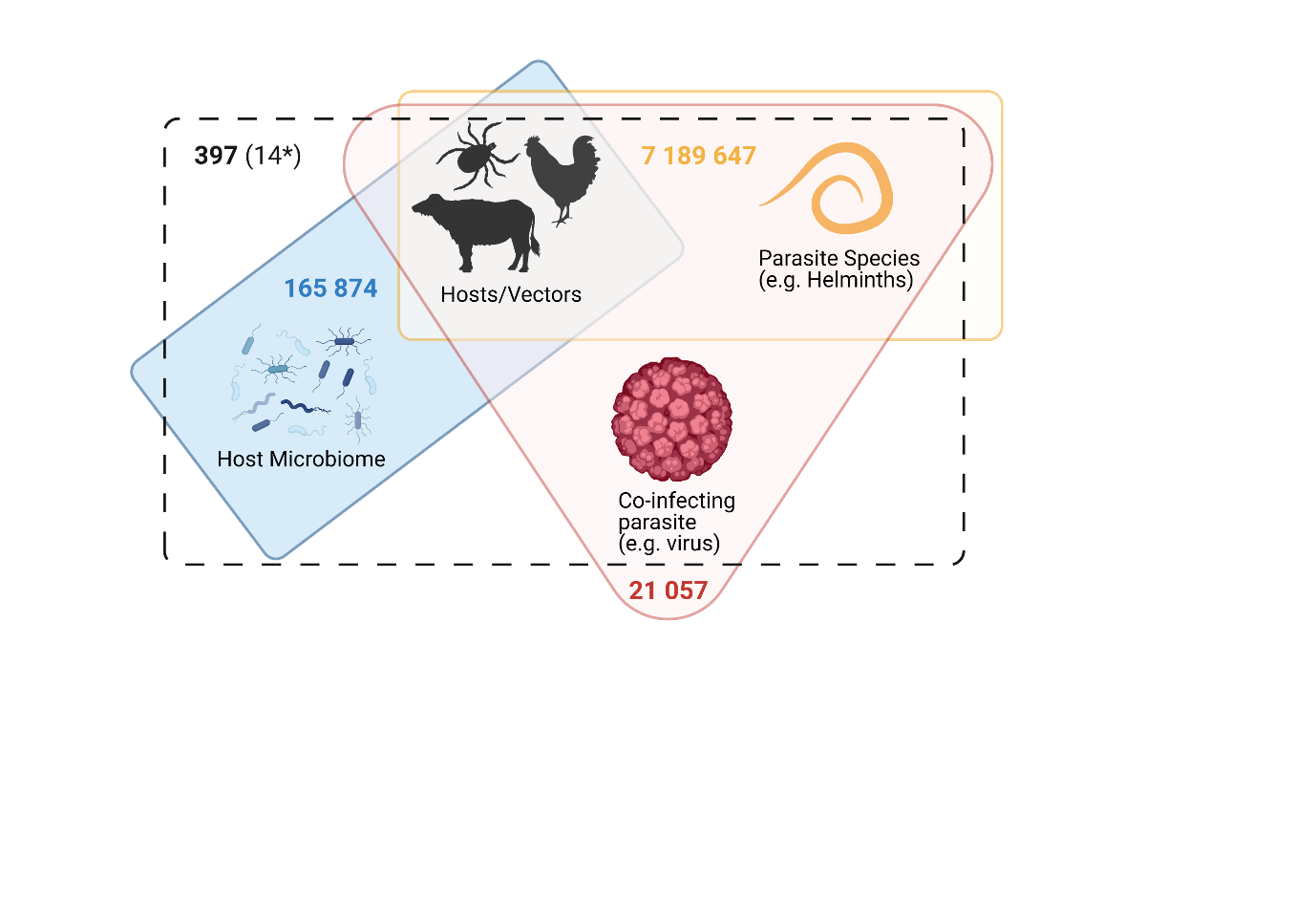
**

**Figure S1. Literature search results for Set #1 (yellow), Set #2 (red), Set #3 (black dashed) and Set #4 (blue).** Numbers correspond to primary research articles yielded by each search, but screening was only done for Set #3. *A total of 14 primary research articles detailing the impact of co-infections on a level of microbiome dysbiosis were uncovered.

**Additional Tables**

**Additional Table S1.** Reference list of the 14 studies included in the systematic review.

| 1 | **Leite, F.L.L.**, Singer, R.S., Ward, T., Gebhart, C.J., and Isaacson, R.E. (2018). Vaccination against *Lawsonia intracellularis* decreases shedding of *Salmonella enterica* serovar Typhimurium in co-infected pigs and alters the gut microbiome. Sci. Rep. *8,* DOI:10.1038/s41598-018-21255-7. |
| --- | --- |
| 2 | **Abdelhamid, M.K.**, Quijada, N.M., Dzieciol, M., Hatfaludi, T., Bilic, I., Selberherr, E., Liebhart, D., Hess, C., Hess, M., and Paudel, S. (2020). Co-infection of chicken layers with *Histomonas meleagridis* and avian pathogenic *Escherichia coli* is associated with dysbiosis, cecal colonization and translocation of the bacteria from the gut lumen. Front. Microbiol. 11, doi: 10.3389/fmicb.2020.586437. |
| 3 | **Wan, X.**, Xu, L., Sun, X., Li, H., Yan, F., Han, R., Li, H., Li, Z., Tian, Y., Liu, X., et al. (2020). Gut microbiota profiles of commercial laying hens infected with tumorigenic viruses. BMC Vet. Res. *16*, doi.org/10.1186/s12917-020-02430-3. |
| 4 | **Whary, M.T.**, Muthupalani, S., Ge, Z., Feng, Y., Lofgren, J., Shi, H.N., Taylor, N.S., Correa, P., Versalovic, J., Wang, T.C., et al. (2014). Helminth co-infection in *Helicobacter pylori* infected INS-GAS mice attenuates gastric premalignant lesions of epithelial dysplasia and glandular atrophy and preserves colonization resistance of the stomach to lower bowel microbiota. Microbes Infect. *16*, 345–355. |
| 5 | **Osborne, L.C.**, Monticelli, L.A., Nice, T.J., Sutherland, T.E., Siracusa, M.C., Hepworth, M.R., Tomov, V.T., Kobuley, D., Tran, S. V., Bittinger, K., et al. (2014). Virus-helminth coinfection reveals a microbiota-independent mechanism of immunomodulation. Science (80-. ). *345*, 578–582. |
| 6 | **Bartelt, L.A.**, Bolick, D.T., Mayneris-Perxachs, J., Kolling, G.L., Medlock, G.L., Zaenker, E.I., Donowitz, J., Thomas-Beckett, R.V., Rogala, A., Carroll, I.M., et al. (2017). Cross-modulation of pathogen-specific pathways enhances malnutrition during enteric co-infection with *Giardia lamblia* and enteroaggregative *Escherichia coli*. PLOS Pathog. *13*, e1006471. |
| 7 | **Wang, G.**, He, Y., Jin, X., Zhou, Y., Chen, X., Zhao, J., Zhang, H., and Chen, W. (2018). The effect of co-infection of food-borne pathogenic bacteria on the progression of Campylobacter jejuni Infection in mice. Front. Microbiol. *9*, doi: 10.3389/fmicb.2018.01977. |
| 8 | **Xu, F.**, Cheng, R., Miao, S., Zhu, Y., Sun, Z., Qiu, L., Yang, J., and Zhou, Y. (2020). Prior *Toxoplasma gondii* infection ameliorates liver fibrosis induced by *Schistosoma japonicum* through inhibiting th2 response and improving balance of intestinal flora in mice. Int. J. Mol. Sci. *21*. |
| 9 | **Youmans, B.P.**, Ajami, N.J., Jiang, Z.-D., Campbell, F., Wadsworth, D., Petrosino, J.F., Dupont, H.L., and Highlander, S.K. (2015). Characterization of the human gut microbiome during travelers’ diarrhea. Gut Microbes *6*, 110–119. |
| 10 | **Mathew, S.**, Smatti, M.K., Al Ansari, K., Nasrallah, G.K., Al Thani, A.A., and Yassine, H.M. (2019). Mixed Viral-Bacterial Infections and Their Effects on Gut Microbiota and Clinical Illnesses in Children. Sci. Rep. *9*, 1–12. |
| 11 | **Easton, A. V.**, Raciny-Aleman, M., Liu, V., Ruan, E., Marier, C., Heguy, A., Yasnot, M.F., Rodriguez, A., and Loke, P. (2020). Immune response and microbiota profiles during coinfection with *Plasmodium vivax* and soil-transmitted helminths. MBio *11*, 1–17. |
| 12 | **Mejia, R.**, Damania, A., Jeun, R., Bryan, P.E., Vargas, P., Juarez, M., Cajal, P.S., Nasser, J., Krolewiecki, A., Lefoulon, E., et al. (2020). Impact of intestinal parasites on microbiota and cobalamin gene sequences: A pilot study. Parasites and Vectors *13*, 200. |
| 13 | **Taylor, B.C.**, Weldon, K.C., Ellis, R.J., Franklin, D., Groth, T., Gentry, E.C., Tripathi, A., McDonald, D., Humphrey, G., Bryant, M., et al. (2020). Depression in Individuals Coinfected with HIV and HCV Is Associated with Systematic Differences in the Gut Microbiome and Metabolome. MSystems *5*, 1–16. |
| 14 | **Sabey, K.A.**, Song, S.J., Jolles, A., Knight, R., and Ezenwa, V.O. (2021). Coinfection and infection duration shape how pathogens affect the African buffalo gut microbiota. ISME J. *15*, 1359–1371. |

**Additional Table S2.** Pair-wise comparisons of PERMDISP results comparing the gut microbial composition of uninfected, singly infected and co-infected individuals using A) weighted UniFrac and B) unweighted UniFrac distances. Only p-values are shown.

| **A)** | **Uninfected** | **Helminth+** | **AdV+** | **Co-infected** |
| --- | --- | --- | --- | --- |
| **Uninfected** |  | 0.014 | 0.030 | 0.027 |
| **Helminth+** | 0.016 |  | 0.546 | 0.452 |
| **AdV+** | 0.027 | 0.537 |  | 0.847 |
| **Co-infected** | 0.025 | 0.441 | 0.830 |  |
| **B)** |  |  |  |  |
| **Uninfected** |  | 0.144 | 0.012 | 0.002 |
| **Helminth+** | 0.142 |  | 0.172 | 0.016 |
| **AdV+** | 0.010 | 0.179 |  | 0.069 |
| **Co-infected** | 0.000 | 0.016 | 0.078 |  |
